# Prognostic significance of CK7, CK20, and CDX2 expression in small intestinal adenocarcinoma

**DOI:** 10.1016/j.esmogo.2026.100346

**Published:** 2026-06-18

**Authors:** P.J.M. Schafrat, J.D.G. Linssen, T.R. de Back, L.E. Nijman, M.C.S. van der Heijden, N.C.T. van Grieken, G. Kazemier, P. Drillenburg, M. Chalabi, F.N. van Erning, I.H.J.T. de Hingh, R.S.A. Goedegebuure, D.W. Sommeijer, A. Farina Sarasqueta, L. Vermeulen

**Affiliations:** 1 Laboratory of Experimental Oncology and Radiobiology, Cancer Center Amsterdam, Amsterdam UMC and University of Amsterdam, Amsterdam, The Netherlands; 2 Oncode Institute, Utrecht, The Netherlands; 3 Amsterdam Gastroenterology Endocrinology Metabolism, Amsterdam, The Netherlands; 4 Departments of Medical Oncology, Amsterdam UMC, Amsterdam, The Netherlands; 5 Departments of Gastroenterology, Amsterdam UMC, Amsterdam, The Netherlands; 6 Cancer Center Amsterdam, Amsterdam UMC, University of Amsterdam, Amsterdam, The Netherlands; 7 Departments of Pathology, Amsterdam UMC, Amsterdam, The Netherlands; 8 Departments of Surgery, Amsterdam UMC, Amsterdam, The Netherlands; 9 Department of Pathology, OLVG, Amsterdam, The Netherlands; 10 Netherlands Cancer Institute, Department of Gastrointestinal Oncology, Amsterdam, The Netherlands; 11 Department of Research and Development, Netherlands Comprehensive Cancer Organization (IKNL), Utrecht, The Netherlands; 12 Department of Surgery, Catharina Hospital, Eindhoven, The Netherlands; 13 Department of Epidemiology, GROW-School for Oncology and Developmental Biology, Maastricht University, Maastricht, The Netherlands; 14 Department of Internal Medicine, Meander Medical Center, Amersfoort, The Netherlands; 15 Department of Internal Medicine, Flevo Hospital, Almere, The Netherlands

**Keywords:** caudal-type homeodomain transcription factor 2 (CDX2) expression profiles, cytokeratin 7 (CK7), cytokeratin 20 (CK20), prognostic value, small intestinal adenocarcinoma

## Abstract

**Background:**

Small intestinal adenocarcinoma (SIA) is a rare gastrointestinal malignancy for which prognostic biomarkers remain poorly defined. Immunohistochemical markers cytokeratin 7 (CK7), cytokeratin 20 (CK20), and caudal-type homeobox transcription factor 2 (CDX2) are diagnostic markers that are prognostic in other intestinal cancers, but their prognostic significance in SIA has not been studied before.

**Patients and methods:**

This nationwide, retrospective, population-based cohort study used data from the Netherlands Cancer Registry and the Dutch pathology database. Patients diagnosed with primary SIA between 1999 and 2019 and with registered CK7, CK20, or CDX2 expression levels were included. Overall survival (OS) was analyzed using Kaplan–Meier curves and multivariable Cox regression models adjusted for age, sex, stage, tumor location, and treatment. The findings were validated in a detailed cohort of 128 SIA patients.

**Results:**

Of 2697 patients diagnosed with SIA, expression data were available for 691 patients. CK7 positivity was associated with more proximal, advanced, and microsatellite stable tumors. CK7-negative, CK20-positive, and CDX2-positive tumors demonstrated significantly improved OS (all *P* ≤ 0.002). In multivariable analysis, lack of CK20 and CDX2 expression were independent predictors of poorer survival [hazard ratio (HR) 1.37, 95% confidence interval (CI) 1.02-1.83 and HR 1.51, 95% CI 1.06-2.17, respectively]. Combined expression profiles identified distinct prognostic subgroups, which were confirmed in the validation cohort.

**Conclusion:**

CK20 and CDX2 expression are independent prognostic biomarkers in SIA, regardless of primary tumor location. Their assessment may improve risk stratification and refine disease classification, bridging diagnostic and prognostic utility in this rare cancer type.

## Highlights

• CK20 and CDX2 are independent prognostic biomarkers in SIA.
• CK7 positivity identifies a clinically relevant high-risk subgroup in SIA.
• Routine assessment of these immunohistochemical markers enables fast and cost-effective risk stratification.

## Introduction

Small intestinal adenocarcinoma (SIA) is a rare malignancy arising from the epithelial lining of the duodenum, jejunum, or ileum, accounting for only a small fraction of gastrointestinal cancers despite the large surface area of the small intestine. Current estimates suggest an incidence of ∼1.3 per 100 000 person-years.^1^^,^^2^ Of these cases, 12% can be attributed to genetic and nongenetic predisposing conditions.^3^ Due to its rarity, the clinical management of SIA largely follows colorectal cancer (CRC) guidelines, yet overall survival (OS) remains poor, underscoring the importance for disease-specific insights into its biology and prognosis.^4^

In gastrointestinal oncology, immunohistochemical (IHC) markers such as cytokeratin 7 (CK7), cytokeratin 20 (CK20), and caudal-type homeodomain transcription factor 2 (CDX2) are widely used diagnostic tools that help distinguish between tumor origins.^5^, ^6^, ^7^, ^8^, ^9^ CK7 is typically expressed in glandular epithelia of the lung, breast, and upper gastrointestinal tract, whereas CK20 expression predominates in the lower gastrointestinal and urothelial epithelia. CDX2, a transcription factor critical for intestinal differentiation, is widely expressed in colorectal and small intestinal epithelium.^9^

Beyond their diagnostic roles, these markers have been implicated in tumor differentiation and prognosis across multiple cancer types.^10^, ^11^, ^12^, ^13^, ^14^ For example, loss of CDX2 expression has been associated with poor outcomes in CRC and gastric cancer, while CK7 expression has shown variable prognostic implications depending on tumor origin.^12^^,^^15^ However, the prognostic relevance of CK7, CK20, and CDX2 in SIA has not been systematically investigated.

This study aimed to evaluate the prognostic value of CK7, CK20, and CDX2 expression, individually and in combination, in a nationwide cohort of patients with SIA, using data from the Netherlands Cancer Registry (NCR) and the Dutch nationwide pathology database (PALGA), and to validate these findings in a multicenter cohort.

## Patients and methods

This nationwide, retrospective, population-based cohort study combined clinical and pathological data from the NCR and PALGA to identify all patients diagnosed with a primary SIA between 1999 and 2019 with recorded expression levels of CK7, CK20, or CDX2. The origin of the tumor was based on the International Classification of Diseases for Oncology (ICD-O), and only ICD-O code C17 was included in this study. Tumors originating outside the small intestine, including ampullary tumors, were excluded. Clinical information obtained from the NCR included patient characteristics [sex, age at diagnosis, and performance status according to the World Health Organization scale (since 2015 if available)], tumor features [primary tumor location, metastatic sites (since 2009)], clinical TNM (tumor–node–metastasis) stage, microsatellite stability status (if available), and treatment data [surgical resection (yes/no) and systemic therapy (yes/no)]. Corresponding pathology reports were retrieved from PALGA, which provided histopathological data, including IHC expression profiles, pathological TNM stage, tumor differentiation grade and if available, microsatellite stability status. In both datasets, the used edition of TNM-staging followed the recommendation of the International Union Against Cancer during the period of diagnosis. Both datasets were linked through unique anonymized case identifiers. Only patients with available expression data for at least one of the three markers (CK7, CK20, or CDX2) were included.

After completion of the registry-based analyses, a validation cohort was established. This validation cohort included a subset of 128 SIA samples diagnosed between 2000 and 2020, from four Dutch hospitals: OLVG, Catharina Hospital Eindhoven, Netherlands Cancer Institute, and Amsterdam UMC. Detailed clinical and pathological data were retrieved from electronic patient records (by PS and TdB). Tumor slides were stained for CK7, CK20, and CDX2 by IHC, and expression was evaluated by two gastrointestinal pathologists (AFS and MvdH).

### Interpretation of the expression profiles

For interpretation of data from the pathology records, a cut-off based on minimal positive documentation was applied. For IHC staining, four semiquantitative scoring categories were used: 0 = negative (no expression); 1 = mostly negative (weak or <30% of tumor cells positive); 2 = mostly positive (moderate or >30% of tumor cells positive); and 3 = positive (strong diffuse expression). Scores of 0-1 were classified as negative and 2-3 as positive.

### Statistical analyses

Descriptive statistics were used to summarize patient and tumor characteristics. Categorical variables were expressed as frequencies and proportions, while continuous variables were summarized as means with standard deviations or medians with interquartile ranges, depending on distribution. Comparisons between groups were made using the chi-square test for categorical variables and the Kruskal–Wallis test for continuous variables.

OS, measured from date of diagnosis, was visualized using Kaplan–Meier curves and compared using the log-rank test, excluding subgroups with <10 patients for clarity. Subgroup analyses were carried out in patients with local (stage I-III) versus metastatic (stage IV) disease, per primary tumor location (duodenum, jejunum, or ileum), and in patients with microsatellite stable (MSS)/mismatch repair proficient (pMMR) tumors. Multivariable Cox proportional hazard models were applied to estimate the association between CK7, CK20, and CDX2 expression and OS, adjusted for age, sex, tumor stage, primary location, surgical resection (yes/no), and systemic treatment (yes/no). Due to limited data, performance status, MMR status, and differentiation grade could not be included as covariates. When the proportional hazards assumption was violated, analyses were stratified accordingly, and the pooled hazard ratios were shown. Missing data were reported but not imputed. Cases with missing variables were excluded from multivariable models. A two-sided *P* value <0.05 was considered statistically significant. All analyses were conducted using RStudio, version 4.4.2 [RRID:SCR_001905].

## Results

In total, 2697 patients were diagnosed with SIA in the Netherlands between 1999 and 2019.^4^ Expression profiles of CK7, CK20, or CDX2 were available for 691 patients (Supplementary Table S1, available at https://doi.org/10.1016/j.esmogo.2026.100346). CK7 and CK20 expression levels were reported in 94% of patients (*n* = 651 and *n* = 647, respectively), while CDX2 levels were available for 65% (*n* = 451). In 414 patients (60%), expression levels of all three markers had been registered.

### Patient characteristics

Clinicopathological characteristics from the patients in the nationwide cohort are summarized in Table 1. Overall, no differences in sex were found, the duodenum was seen as the most frequently affected tumor location (59%), and SIA was regularly diagnosed between the age of 50-75 years. Advanced stage at diagnosis was found in 47% of patients, with mostly single-organ metastases (63%). Most tumors were moderately (48%) or poorly differentiated (48%). In 28% of the patients, tumors were microsatellite instable (MSI)/mismatch repair deficient (dMMR).

**Table 1:** Characteristics of patients diagnosed with a primary malignancy of the small intestine per expression profile (NCR)

	All patients (*n* = 691) *n* (%)	CK7 positive (*n* = 369) *n* (%)	CK7 negative (*n* = 282) *n* (%)	CK20 positive (*n* = 429) *n* (%)	CK20 negative (*n* = 218) *n* (%)	CDX2 positive (*n* = 375) *n* (%)	CDX2 negative (*n* = 76) *n* (%)
Sex							
Male	351 (51)	188 (51)	141 (50)	209 (49)	117 (54)	197 (53)	38 (50)
Female	340 (49)	181 (49)	141 (50)	220 (51)	101 (46)	178 (47)	38 (50)
*P*		0.872		0.268		0.782	
Age category at diagnosis (years)							
Median [IQR]	66 [58-75]	67 [58-76]	66 [58-74]	66 [57-75]	68 [60-75]	68 [59-75]	68 [60-74]
<50	90 (13)	44 (12)	37 (13)	65 (15)	19 (9)	46 (12)	2 (3)
50-75	442 (64)	232 (63)	184 (65)	265 (62)	148 (68)	236 (63)	57 (75)
>75	159 (23)	93 (25)	61 (22)	99 (23)	51 (23)	93 (25)	17 (22)
*P*		0.614		0.105		0.516	
Period of diagnosis							
1999-2006	86 (12)	43 (12)	36 (13)	57 (13)	26 (12)	5 (1)	4 (5)
2007-2013	247 (36)	143 (39)	89 (32)	152 (35)	76 (35)	100 (27)	29 (38)
2014-2019	358 (52)	183 (50)	157 (56)	220 (51)	116 (53)	270 (72)	43 (57)
*P*		0.164		0.850		**0.007**	
Tumor location							
Duodenum	406 (59)	240 (65)	146 (52)	234 (55)	139 (64)	230 (61)	46 (61)
Jejunum	150 (22)	62 (17)	79 (28)	107 (25)	37 (17)	82 (22)	14 (18)
Ileum	92 (13)	45 (12)	36 (13)	60 (14)	27 (12)	40 (11)	12 (16)
Overlapping	13 (2)	7 (2)	6 (2)	8 (2)	5 (2)	7 (2)	2 (3)
NOS	30 (4)	15 (4)	15 (5)	20 (5)	10 (5)	16 (4)	2 (3)
*P*		**0.006**		0.155		0.662	
Stage							
I	15 (2)	5 (2)	10 (4)	8 (2)	7 (3)	8 (2)	2 (3)
II	168 (26)	79 (23)	77 (29)	102 (25)	52 (26)	90 (26)	19 (27)
III	159 (25)	76 (22)	75 (28)	106 (26)	42 (21)	98 (28)	15 (21)
IV	304 (47)	182 (53)	104 (39)	185 (46)	102 (50)	155 (44)	35 (49)
Missing	45	27	16	28	15	24	5
*P*		**0.006**		0.483		0.833	
Grade of differentiation							
Well differentiated	21 (5)	12 (5)	9 (5)	12 (4)	8 (5)	11 (5)	2 (4)
Moderately differentiated	223 (48)	104 (43)	107 (54)	162 (57)	50 (33)	126 (52)	16 (31)
Poorly differentiated	223 (48)	126 (52)	82 (41)	110 (39)	95 (62)	105 (43)	34 (65)
Missing	224	127	84	145	65	133	24
*P*		0.066		**<0.001**		**0.015**	
MMR status^a^							
MSS	186 (72)	87 (81)	90 (65)	134 (76)	47 (69)	123 (69)	17 (85)
MSI	73 (28)	20 (19)	48 (35)	43 (24)	21 (31)	55 (31)	3 (15)
Missing	432	262	144	252	150	197	56
*P*		**0.008**		0.374		0.222	
Site of metastases^b^							
Single	191 (63)	121 (66)	59 (57)	113 (61)	67 (66)	94 (61)	26 (74)
Two	75 (25)	41 (23)	31 (30)	48 (26)	22 (22)	46 (30)	7 (28)
Three or more	26 (9)	16 (9)	7 (7)	16 (9)	9 (9)	10 (6)	2 (8)
*P*		NA		NA		NA	
OS (months)							
Median [95% CI]	11.8 [10.4-15.2]	8.7 [7.1-10.3]	22.6 [17.2-33.9]	16.6 [13.7-21.6]	7.9 [5.6-9.5]	13.9 [11.0-19.1]	4.8 [3.2-10.1]
*P*		**<0.001**		**<0.001**		**0.002**	

CDX2, caudal-type homeodomain transcription factor 2; CI, confidence interval; CK, cytokeratin; IQR, interquartile range; MMR, mismatch repair; MSI, microsatellite instable; MSS, microsatellite stable; NCR, Netherlands Cancer Registry; NOS, not otherwise specified; OS, overall survival.

Bold values are statistically significant.

a Percentages are based on the number of patients with known MMR status (*n* = 259) for CK7 positive (*n* = 107), CK7 negative (*n* = 138), CK20 positive (*n* = 177), CK20 negative (*n* = 68), CDX2 positive (*n* = 178), and CDX2 negative (*n* = 20).

b Percentages are based on the number of patients with registered metastases.

CK7-positive tumors were more frequently located in the proximal small intestine, presented at a more advanced stage, were more likely to have poor differentiation, and were predominantly MSS/pMMR compared with CK7-negative tumors. Poor differentiation was the only significant difference between CK20-positive and CK20-negative tumors (*P* < 0.001) and CDX2-positive versus CDX2-negative tumors (*P* = 0.015).

### Survival

Median OS across all patients was 11.8 months [95% confidence interval (CI) 10.4-15.2] with a 5-year OS of 24% (Figure 1A). OS differed by primary tumor location, stage of disease, and MSS status (Figure 1B-D). Patients with duodenal tumors showed the worst OS compared with patients with jejunal and ileal tumors (*P* < 0.001*).* Patients with earlier stage disease showed a more favorable OS compared to those with advanced stage disease (*P* < 0.001), with 5-year OS differing from 64% for stage I disease, 48% for stage II, 36% for stage III, and 5% for stage IV disease. Patients with MSI/dMMR tumors demonstrated significantly longer OS compared with patients with MSS/pMMR tumors (*P* < 0.001).

**Figure 1:**
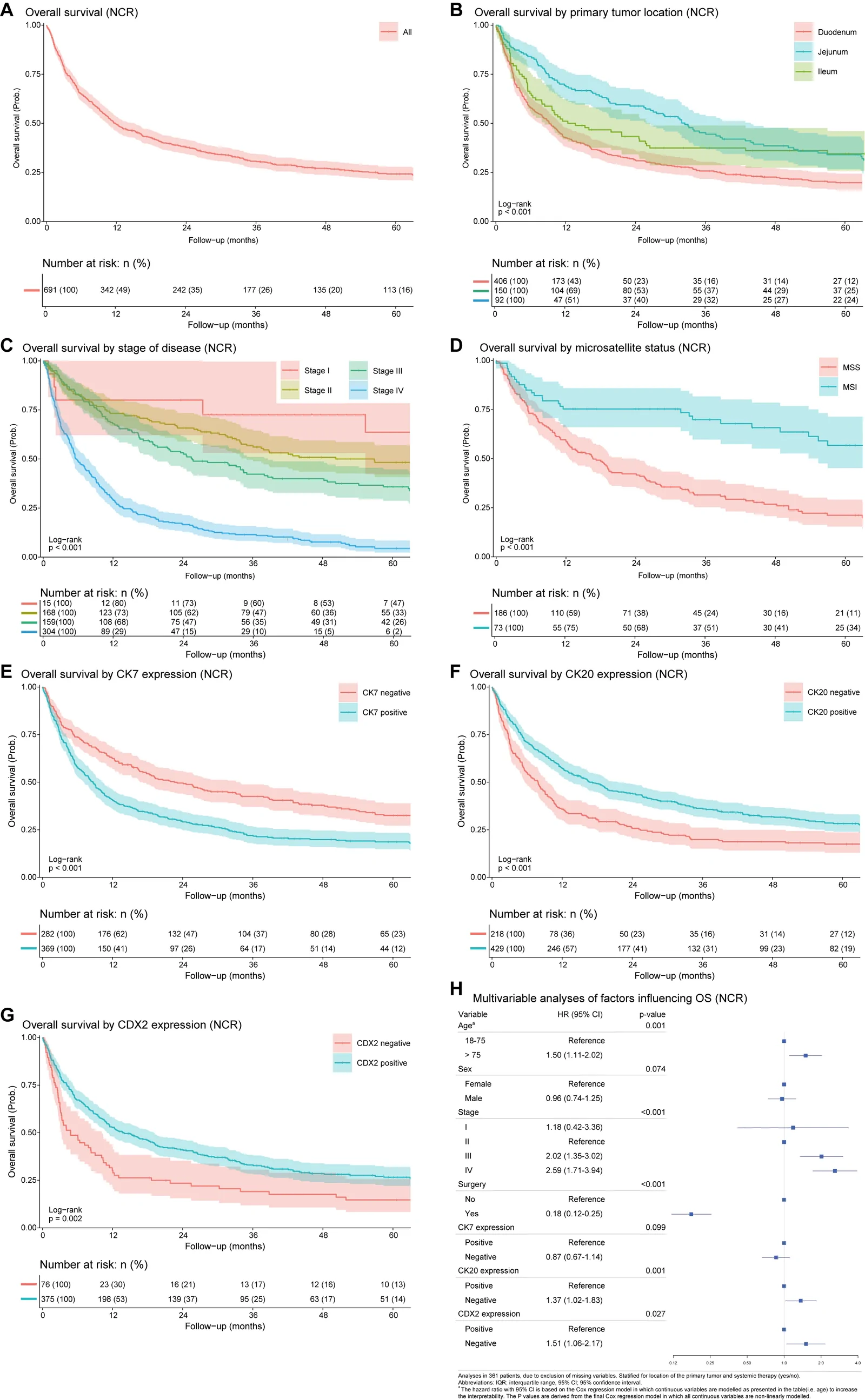
**(A) OS (NCR).** (B) OS by primary tumor location (NCR). (C) OS by stage of disease (NCR). (D) OS by microsatellite status (NCR). (E) OS by CK7 expression (NCR). (F) OS by CK20 expression (NCR). (G) OS by CDX2 expression (NCR). (H) Forest plot multivariable analyses with single expression profiles (NCR). CDX2, caudal-type homeodomain transcription factor 2; CK, cytokeratin; NCR, Netherlands Cancer Registry; OS, overall survival.

Survival analyses of individual markers revealed that patients with CK7-negative tumors demonstrated significantly longer OS compared with those with CK7-positive tumors [median OS (mOS) 22.6 months, 95% CI 17.2-33.9 versus 8.7 months, 95% CI 7.1-10.3, *P* < 0.001; Figure 1E]. Similarly, patients with CK20-positive (mOS 16.6 months, 95% CI 13.7-21.6 versus 7.9 months, 95% CI 5.6-9.5, *P* < 0.001) and CDX2-positive tumors (mOS 13.9 months, 95% CI 11.0-19.1 versus 4.8 months, 95% CI 3.2-10.1, *P* = 0.002) had better survival probability compared with their negative counterparts (Figure 1F and G). Improved survival remained consistent for CK7-negative, CK20-positive, and CDX2-positive tumors across subgroup analyses per primary tumor location, disease stage, or in MSS/pMMR tumors (Supplementary Figures S1-S6, available at https://doi.org/10.1016/j.esmogo.2026.100346). In multivariable Cox regression analyses, stratified for tumor location and systemic treatment (yes/no), older age, advanced stage, absence of surgical resection, lack of CK20 and lack of CDX2 expression were independent predictors of poorer survival (Figure 1H).

### Combined expression profiles

The strong associations with outcome for the expression of individual markers across patient subsets prompted us to assess the markers jointly. Differences were observed in disease stage (*P* < 0.001), primary tumor location (*P* = 0.052), and mOS (*P* < 0.001) (Supplementary Table S2, available at https://doi.org/10.1016/j.esmogo.2026.100346). Patients with CK7-negative, CDX2-positive tumors, irrespective of CK20 status, showed the most favorable OS (Figure 2A). The relatively worst OS was seen in patients with CK7-positive, CK20-negative tumors, independent of CDX2-expression (Figure 2A).

**Figure 2:**
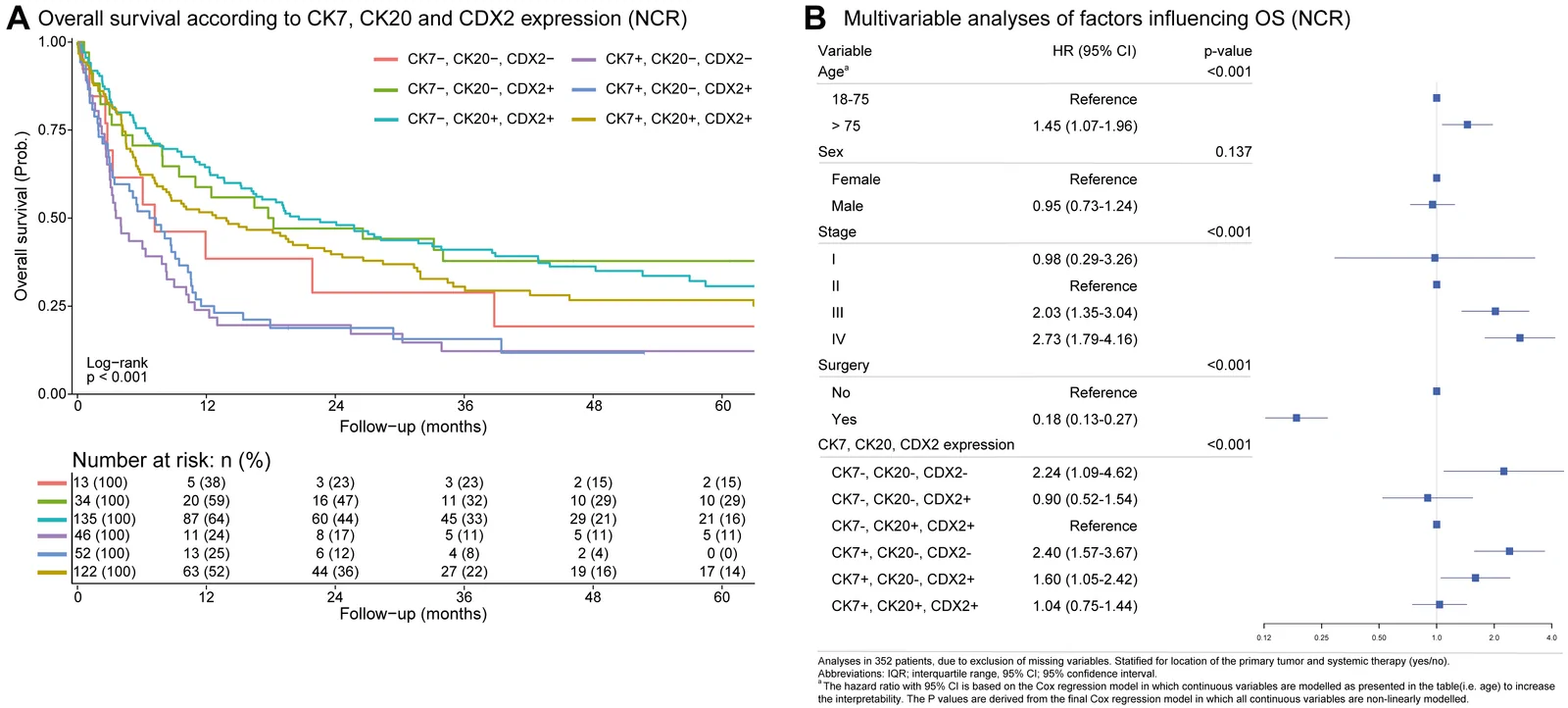
**(A) OS based on CK7, CK20, and CDX2 expression (NCR).** (B) Forest plot multivariable analyses with combined expression profiles (NCR). CDX2, caudal-type homeodomain transcription factor 2; CK, cytokeratin; NCR, Netherlands Cancer Registry; OS, overall survival.

Multivariable analyses, stratified for primary tumor location and systemic treatment (yes/no), showed age, stage, surgical resection, and combined expression profiles to be independent factors for OS (Figure 2B).

### Validation cohort

The nationwide cohort data allowed to assess the expression of markers in a large number of patients, in particular considering the rarity of SIA. However, there are limitations with respect to the interpretation of IHC results from pathology reports and in the clinical data of a nationwide registry. Hence, we sought to validate our findings in a detailed validation set. This multicenter validation cohort contained 128 patients with SIA. All samples were stained by IHC for expression of CK7, CK20, and CDX2 (Figure 3A-F and Supplementary Table S1, available at https://doi.org/10.1016/j.esmogo.2026.100346). Apart from the median OS, no significant clinicopathological differences were observed between expression subgroups (Table 2).

**Figure 3:**
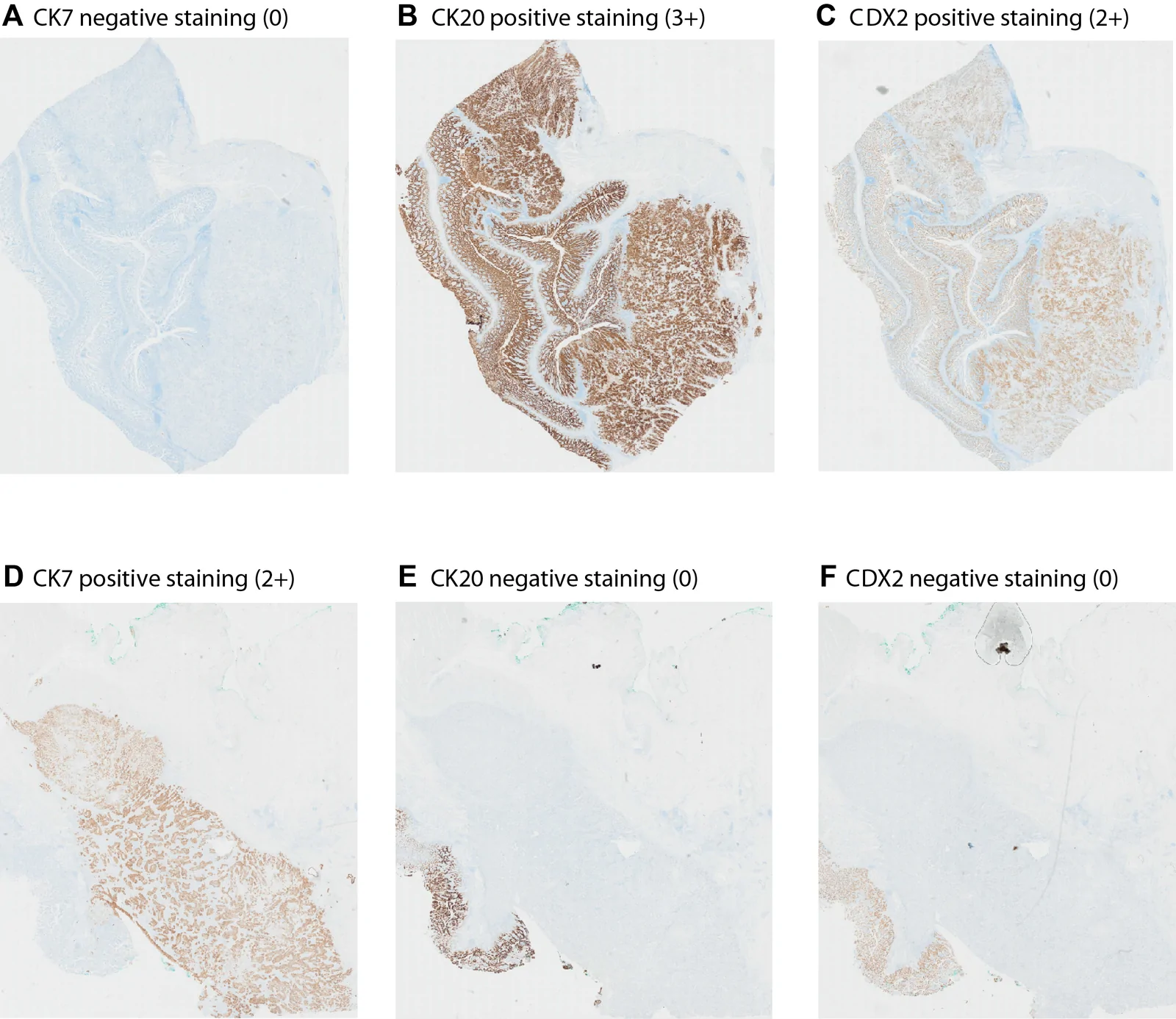
**Immunohistochemical staining.** (A) CK7 negative. (B) CK20 positive. (C) CDX2 positive. (D) CK7 positive. (E) CK20 negative. (F) CDX2 negative. CDX2, caudal-type homeodomain transcription factor 2; CK, cytokeratin.

**Table 2:** Characteristics of patients diagnosed with a primary malignancy of the small intestine per expression profile (validation)

	All patients (*n* = 128) *n* (%)	CK7 positive (*n* = 28) *n* (%)	CK7 negative (*n* = 100) *n* (%)	CK20 positive (*n* = 62) *n* (%)	CK20 negative (*n* = 66) *n* (%)	CDX2 positive (*n* = 84) *n* (%)	CDX2 negative (*n* = 44) *n* (%)
Sex							
Male	56 (44)	20 (71)	47 (47)	32 (52)	24 (36)	40 (48)	16 (36)
Female	72 (56)	8 (29)	52 (52)	30 (48)	42 (64)	44 (52)	28 (64)
*P*		0.106		0.119		0.302	
Age category at diagnosis (years)							
Median [IQR]	67 [59-72]	64 [53-70]	67 [59-73]	66 [59-73]	67 [59-72]	67 [59-73]	66 [60-72]
<50	20 (16)	7 (25)	13 (13)	8 (13)	12 (18)	14 (17)	6 (14)
50-75	83 (65)	17 (61)	66 (66)	43 (69)	40 (61)	54 (64)	29 (66)
>75	25 (20)	4 (14)	21 (21)	11 (18)	14 (21)	16 (19)	9 (20)
*P*		0.271		0.564		0.900	
Tumor location							
Duodenum	91 (71)	24 (86)	67 (60)	39 (63)	52 (79)	55 (66)	36 (82)
Jejunum	25 (20)	2 (7)	23 (23)	16 (26)	9 (14)	21 (25)	4 (9)
Ileum	12 (9)	2 (7)	10 (10)	7 (11)	5 (8)	8 (10)	4 (9)
*P*		0.130		0.133		0.089	
Stage							
I	10 (8)	2 (7)	8 (8)	4 (7)	6 (9)	5 (6)	5 (11)
II	42 (33)	5 (18)	37 (37)	21 (34)	21 (32)	33 (39)	9 (21)
III	56 (44)	15 (54)	41 (41)	27 (44)	29 (44)	35 (42)	21 (48)
IV	20 (16)	6 (21)	14 (14)	10 (16)	10 (15)	11 (13)	9 (21)
*P*		0.259		0.951		0.142	
Mismatch repair status							
Proficient	103 (82)	25 (93)	78 (79)	53 (87)	60 (77)	66 (80)	37 (86)
Deficient	23 (18)	2 (7)	21 (21)	8 (13)	15 (23)	17 (20)	6 (14)
Missing	2	1	1	1	1	1	1
*P*		0.172		0.224		0.512	
Site of metastases^a^							
Single	14 (70)	4 (67)	10 (71)	8 (80)	6 (60)	8 (73)	6 (67)
Two	5 (25)	1 (17)	4 (29)	1 (10)	4 (40)	3 (27)	2 (22)
Three or more	1 (5)	1 (17)	-	1 (10)	-	-	1 (11)
*P*		0.252		0.375		0.456	
OS (months)							
Median [95% CI]	34.1 [26.1-47.3]	19.6 [15.2-38.6]	40.3 [30.2-63.2]	44.9 [34.9-63.2]	25.2 [15.5-34.1]	47.3 [34.1-70.0]	23.4 [15.5-33.0]
*P*		**0.023**		0.210		**<0.001**	

CDX2, caudal-type homeodomain transcription factor 2; CI, confidence interval; CK, cytokeratin; IQR, interquartile range; OS, overall survival.

Bold values are statistically significant.

a Percentages are based on the number of patients with registered metastases.

The results of the cohort study were validated in the validation set. Patients with CK7-negative tumors again showed improved OS compared with those with CK7-positive tumors (mOS 40.4 months, 95% CI 30.2-63.2 versus 19.6 months, 95% CI 15.3-38.6, *P* = 0.023), and patients with CDX2-positive tumors exhibited markedly better survival (mOS 47.3 months, 95% CI 34.1-70.0 versus 23.4 months, 95% CI 15.5-33.0, *P* < 0.001; Table 2 and Figure 4A and C). Also, CK20-positive cancers demonstrated a trend toward a better prognosis (mOS 44.9 months, 95% CI 34.9-63.2 versus 25.2 months, 95% CI 15.5-34.1, *P* = 0.210), in line with our previous results (Table 2 and Figure 4B).

**Figure 4:**
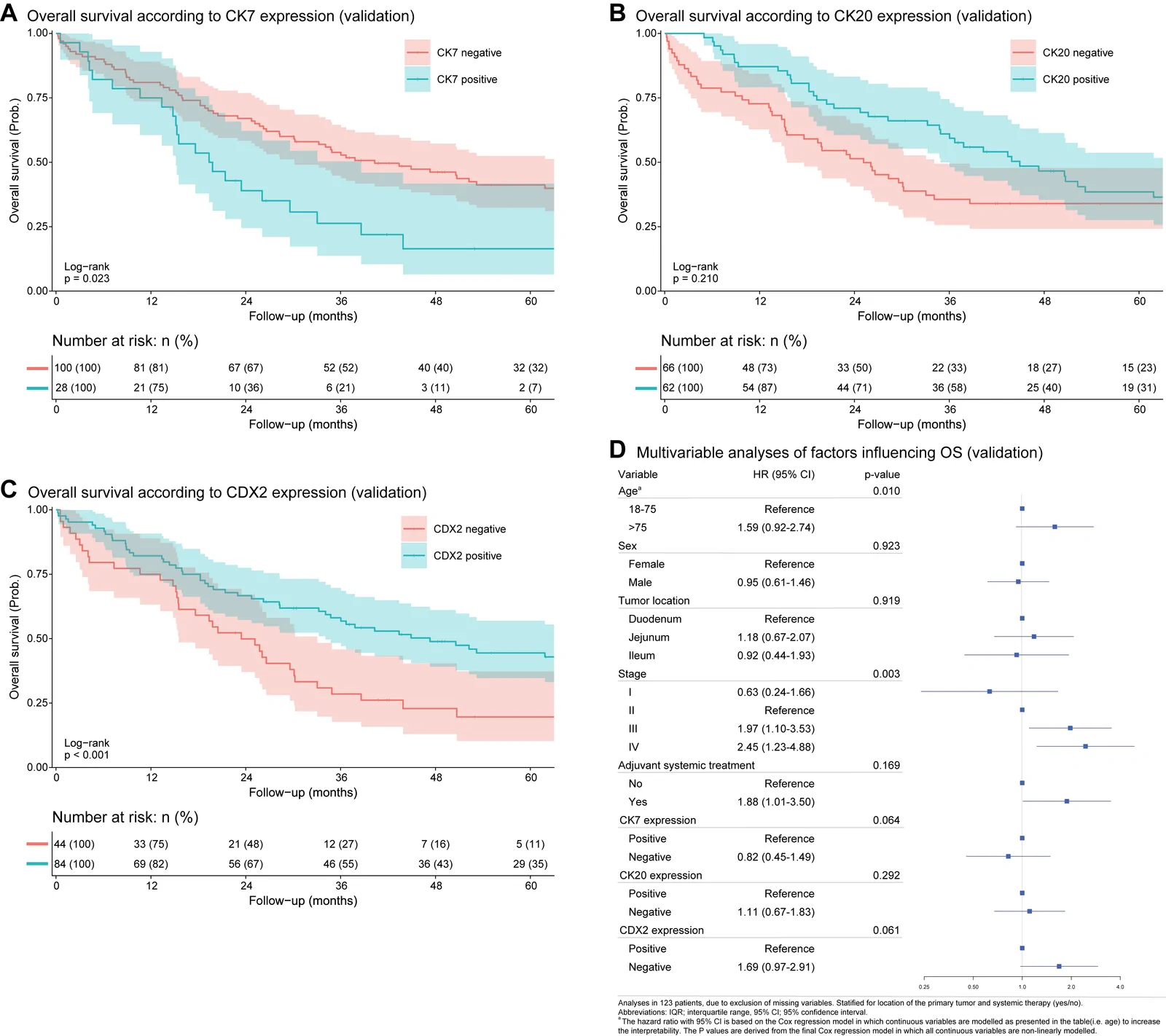
**(A) OS by CK7 expression (validation).** (B) OS by CK20 expression (validation). (C) OS by CDX2 expression (validation). (D) Forest plot multivariable analyses with single expression profiles (validation). CDX2, caudal-type homeodomain transcription factor 2; CK, cytokeratin; OS, overall survival.

In multivariable analyses, younger age and earlier stage were independently associated with longer OS, while CK7 negativity and CDX2 positivity showed a trend toward statistical significance (*P* = 0.064 and *P* = 0.061, respectively, Figure 4D). Also, when analyzed in combination, the three-marker expression profile retained prognostic value, corroborating the results in the cohort study (Figure 5A and B).

**Figure 5:**
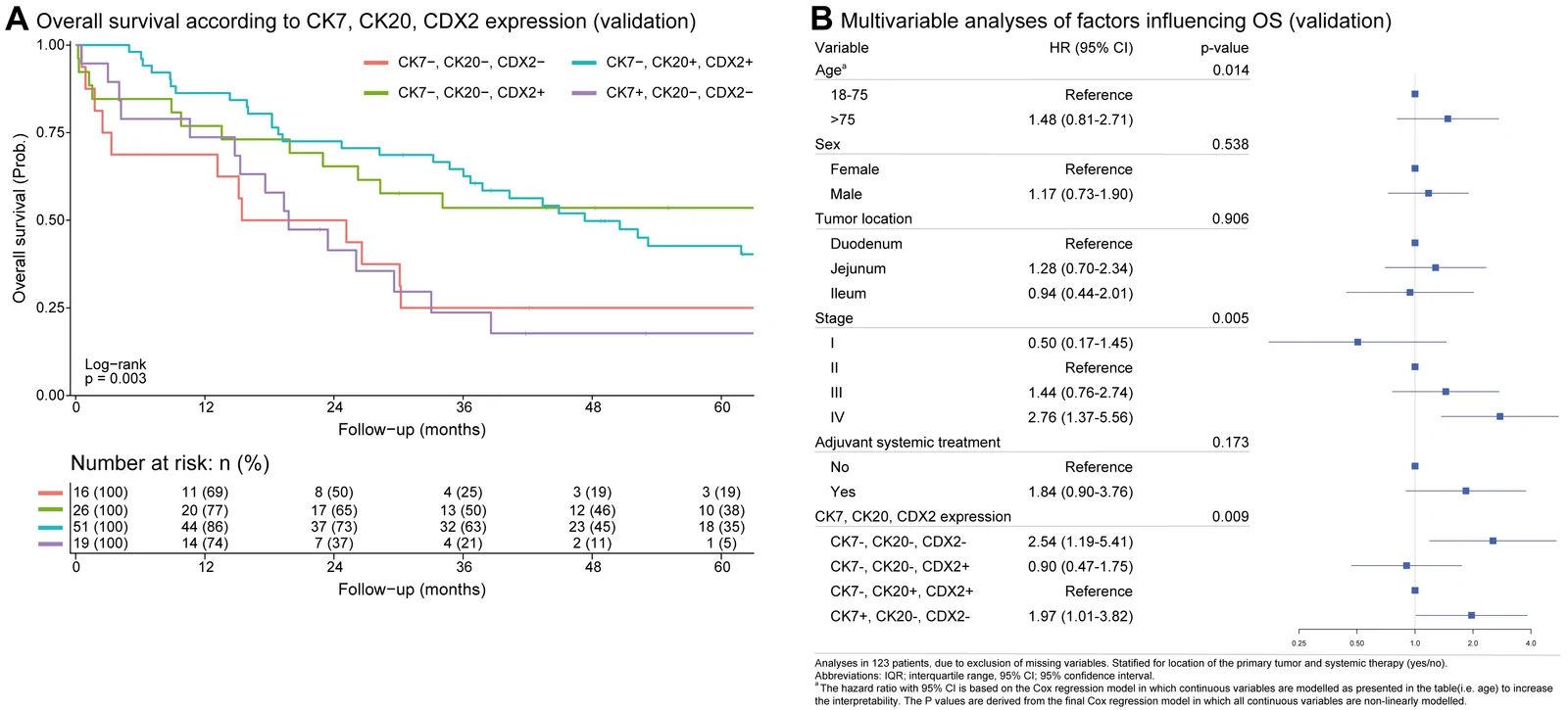
**(A) OS based on CK7, CK20, and CDX2 expression (validation).** (B) Forest plot multivariable analyses with combined expression profiles (validation). CDX2, caudal-type homeodomain transcription factor 2; CK, cytokeratin; OS, overall survival.

## Discussion

In this nationwide population-based study, we evaluated the prognostic relevance of CK7, CK20, and CDX2 expression in SIA. To our knowledge, this is the first large-scale analysis demonstrating that lack of CK20 and CDX2 expression is independently associated with inferior OS in SIA. These findings were robust across disease stage, tumor location, and microsatellite status, and were corroborated in a smaller multicenter validation cohort with more detailed clinical records and a uniform staining protocol. Together, our results suggest that IHC profiling using routinely available markers provides clinically meaningful prognostic information in this rare malignancy.

SIA is often managed according to CRC treatment paradigms, yet its clinical behavior and outcomes are clearly distinct. The expression patterns of CK7, CK20, and CDX2 observed in our cohort place SIA biologically between malignancies of the pancreatic and ampullary region and more distal intestinal tumors, that is, CRCs.^5^^,^^11^^,^^16^, ^17^, ^18^, ^19^ CK7 positivity was observed in a substantial proportion of SIA cases, particularly in duodenal tumors, whereas CK20 and CDX2 expression were less ubiquitous than typically reported in CRC.^11^ This intermediate phenotype mirrors observations in ampullary carcinoma, where CK7, CK20, and CDX2 expression can differentiate between intestinal and pancreatobiliary subtypes and are associated with markedly different prognoses.^19^

Despite this intermediate expression pattern, the prognostic implications of these markers in SIA more closely resemble those described in CRC.^11^^,^^12^^,^^20^ In particular, CK20 and CDX2 positivity were associated with improved survival, whereas CK7 positivity identified a subgroup with distinctly worse outcomes. This dissociation between expression frequency and prognostic behavior underscores that SIA should not be viewed merely as a proximal extension of CRC, but rather as a biologically heterogeneous entity with its own prognostic classifiers.

CK7 expression emerged as a marker of poor prognosis in SIA, consistent with prior reports in CRC in which CK7 positivity has been linked to advanced disease stage, lymph node involvement, and inferior survival.^20^ In our study, CK7-positive tumors were more frequently located in the duodenum, presented at more advanced stages, and were predominantly MSS/pMMR. The markedly shorter median OS of patients with CK7-positive SIA approached that reported for malignancies of the pancreas and ampulla and was substantially worse than outcomes typically observed in CRC.^21^, ^22^, ^23^

These findings suggest that CK7-positive SIA may represent a biologically more aggressive subtype, potentially driven by molecular programs associated with invasiveness and metastatic capacity. Although the mechanistic basis of CK7-associated aggressiveness remains to be fully elucidated, prior studies in CRC have linked CK7 expression to epithelial–mesenchymal transition and enhanced migratory potential.^12^^,^^20^ Our data support the concept that CK7 positivity identifies a high-risk SIA subgroup. This subgroup may benefit from more intensive surveillance and personalized treatment strategies. Specifically, more intense systemic treatment aligned with pancreatic cancer guidelines or those recommended for pancreatobiliary subtypes of ampullary cancers, such as FOLFIRINOX instead of CAPOX/FOLFOX, could be considered for this CK7-positive, high-risk SIA subgroup.

CDX2 is a master regulator of intestinal epithelial differentiation, and its lack of expression has consistently been associated with poor prognosis in CRC and gastric cancer.^11^^,^^17^^,^^18^^,^^24^, ^25^, ^26^ In line with these observations, we demonstrated that CDX2 positivity is strongly associated with improved survival in SIA, independent of stage, tumor location, and treatment. Notably, CDX2-negative tumors did not show clear differences in other clinicopathological parameters, suggesting that loss of CDX2 may capture biological aggressiveness not reflected by conventional staging alone.

Similarly, CK20 positivity was independently associated with improved survival. Although CK20 expression did not correlate strongly with baseline clinicopathological features, its prognostic value became evident in multivariable analyses and in combined expression profiles. Together, these findings support the notion that preservation of an intestinal differentiation program, reflected by CK20 and CDX2 expression, is associated with more favorable tumor biology in SIA.

Assessment of combined CK7, CK20, and CDX2 expression profiles further refined prognostic stratification. Patients with CK7-negative and CDX2-positive tumors, regardless of CK20 status, exhibited the most favorable outcomes, whereas those with CK7-positive and CK20-negative tumors had the poorest survival. These combined profiles remained independently associated with outcome in multivariable analyses and were reproduced in the validation cohort. This combinatorial approach parallels subtype classifications used in ampullary cancer and CRC, where limited IHC panels can capture biologically and clinically relevant heterogeneity.^16^^,^^25^ Our findings suggest that a similar strategy may be feasible in SIA, offering a pragmatic and widely accessible tool for prognostic stratification in routine clinical practice and guide personalized treatment strategies.

The major strength of this study lies in its nationwide scope, long follow-up, and linkage of high-quality cancer registry data with centralized pathology records. The validation in a well-annotated multicenter cohort with standardized IHC assessment further strengthens the credibility of the findings.

Several limitations should be acknowledged. First, the retrospective nature of the study introduces the potential for selection bias, particularly as IHC testing was not carried out uniformly. Furthermore, missing data limited the ability to perform more extensive multivariable analyses (including MMR status and differentiation grade), as well as additional analyses, such as assessing correlations with treatment response. Second, heterogeneity in pathology reporting between diagnostic reporting and research may have influenced classification, although this was mitigated by validation using centrally reviewed samples. Third, the validation cohort was relatively small, which may explain the borderline significance of some associations despite consistent effect sizes. Finally, detailed molecular correlates were not available and warrant investigation in future studies.

Our findings demonstrate that CK20 and CDX2 expression are independent prognostic biomarkers in SIA, while CK7 positivity identifies a high-risk subgroup with poor outcomes. Importantly, currently these markers are routinely assessed in diagnostic pathology, making their implementation for prognostic stratification both feasible and cost-efficient.

Future studies should aim to integrate IHC profiles with molecular data to further elucidate the biological underpinnings of these subtypes and to assess whether they predict response to systemic therapy. Prospective validation will be essential to determine whether CK7, CK20, and CDX2 expression can inform treatment decisions and guide personalized management strategies for patients with this type of malignancy.
