# Supplementary material for: Prognostic significance of CK7, CK20, and CDX2 expression in small intestinal adenocarcinoma
Source: ESMO Gastrointest Oncol. 2026 Jun 18;13(Pt B):100346. doi: 10.1016/j.esmogo.2026.100346 (PMC13712183; doi:10.1016/j.esmogo.2026.100346)

Supplementary figure 1

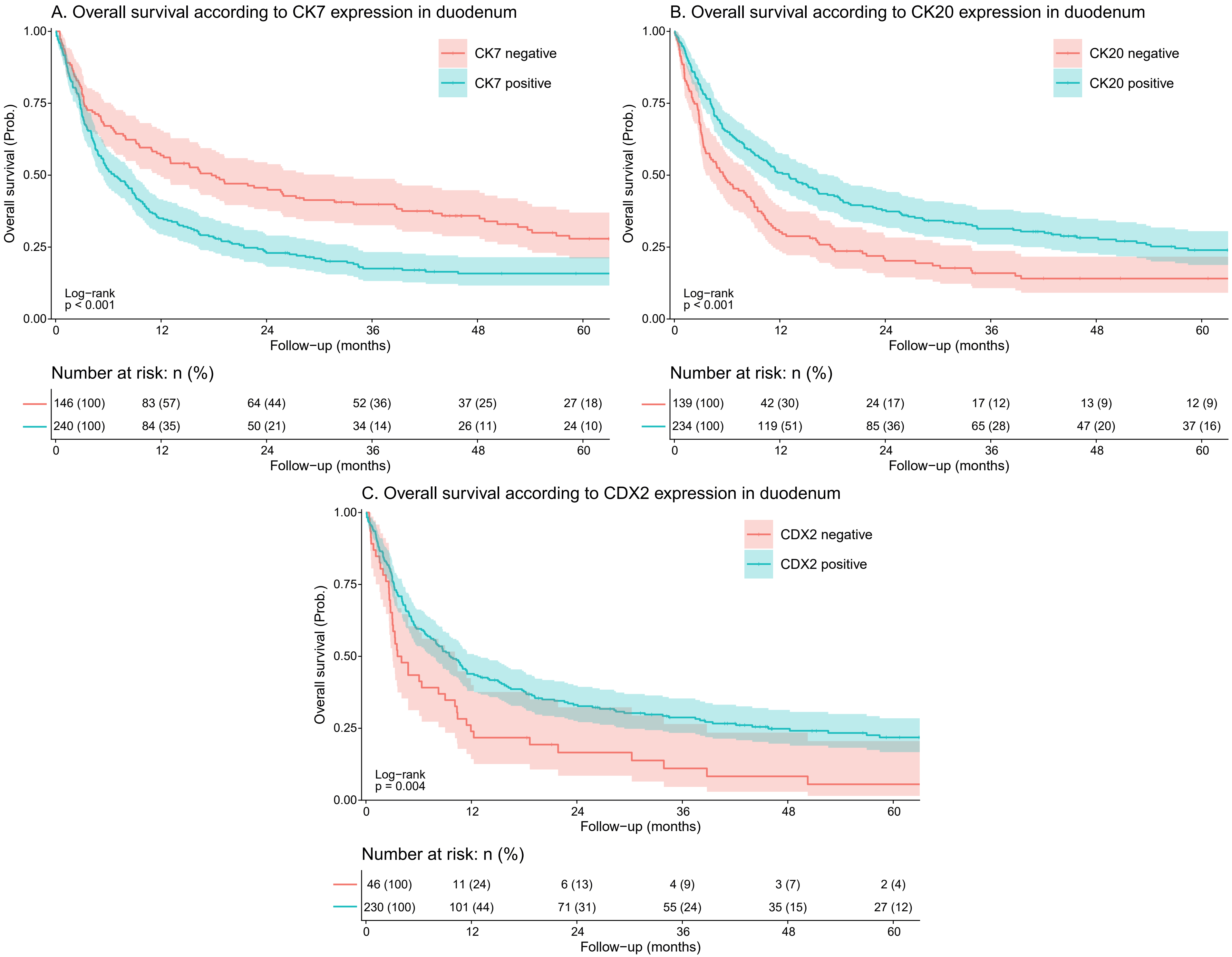

Supplementary figure 2

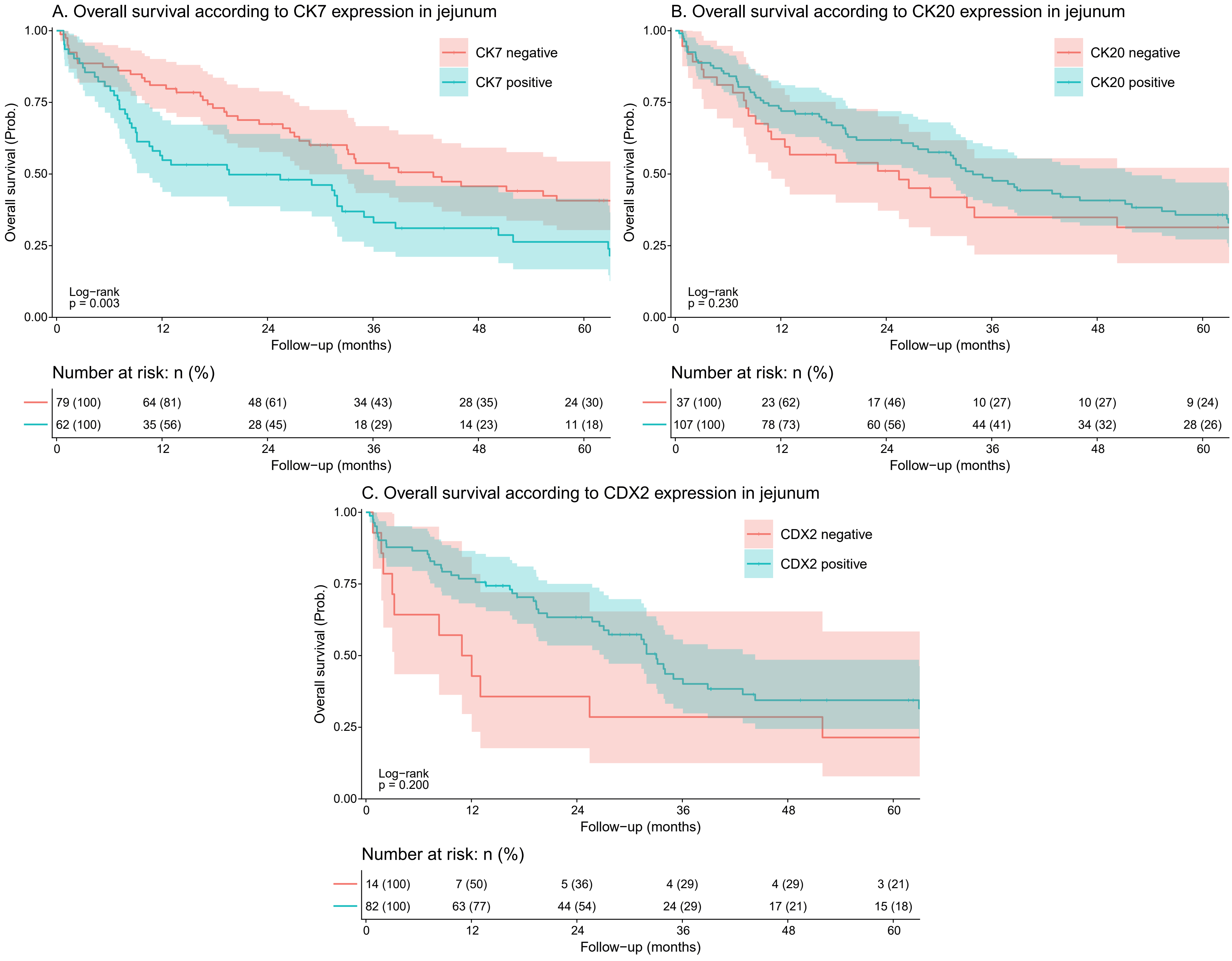

Supplementary figure 3

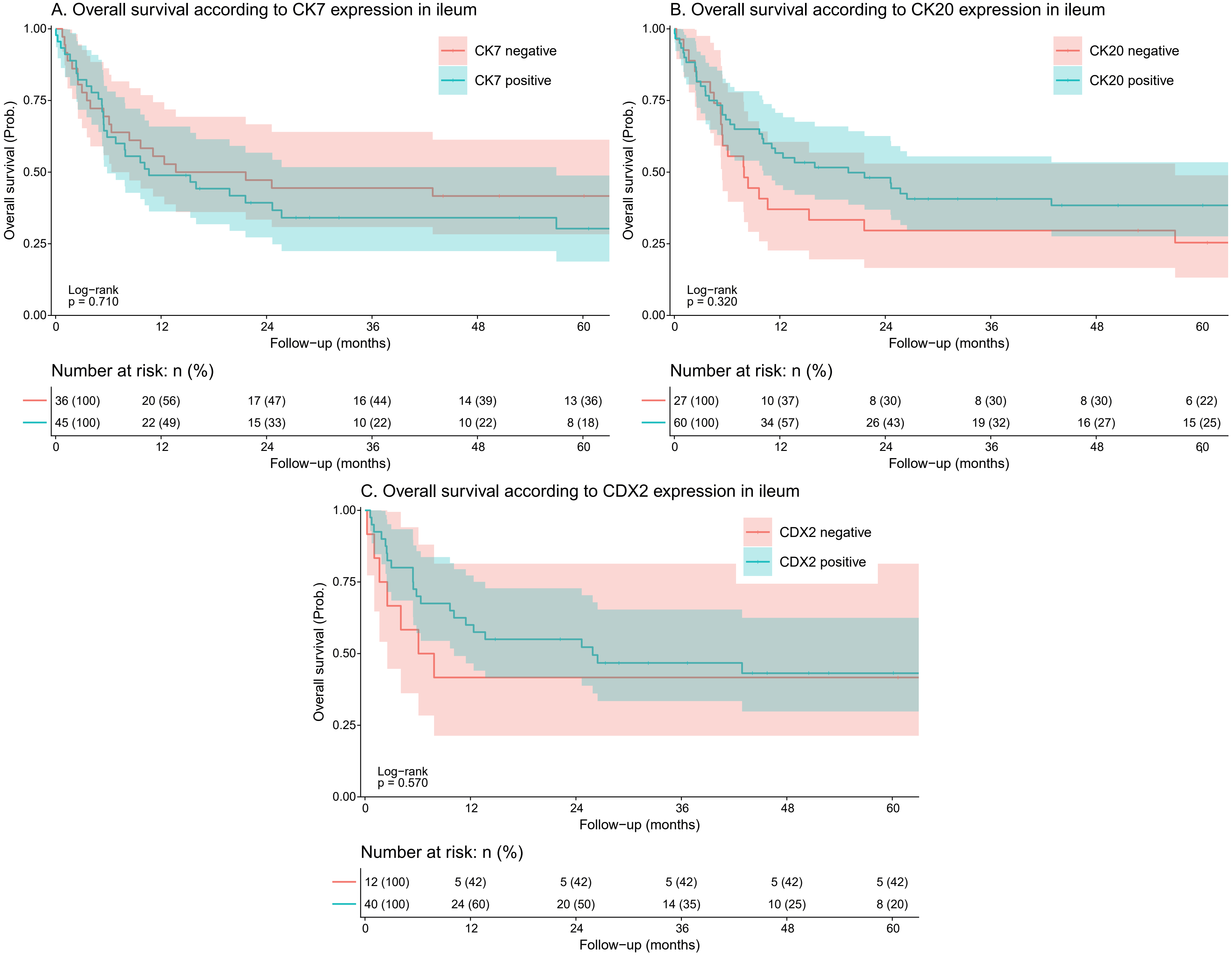

Supplementary figure 4

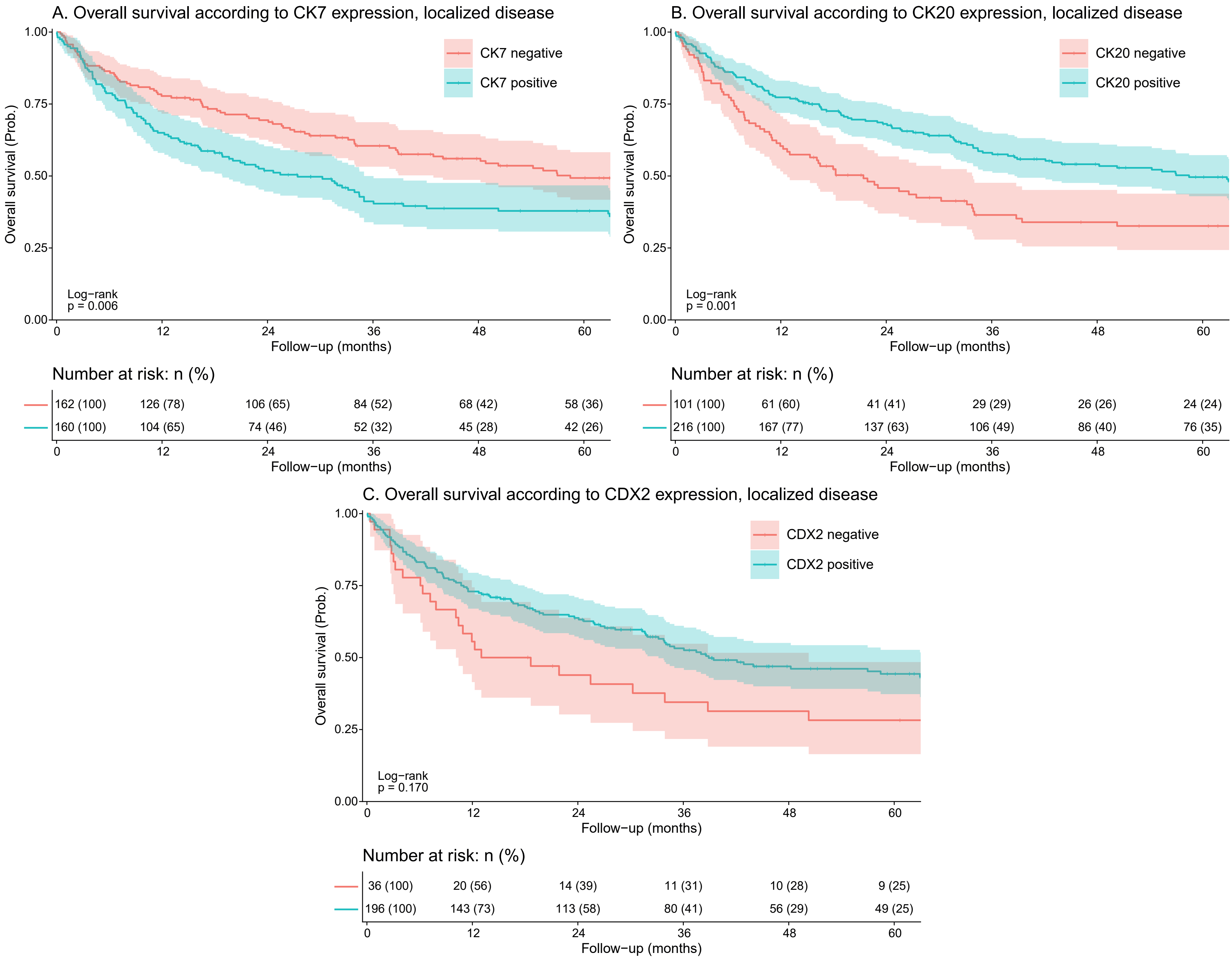

Supplementary figure 5

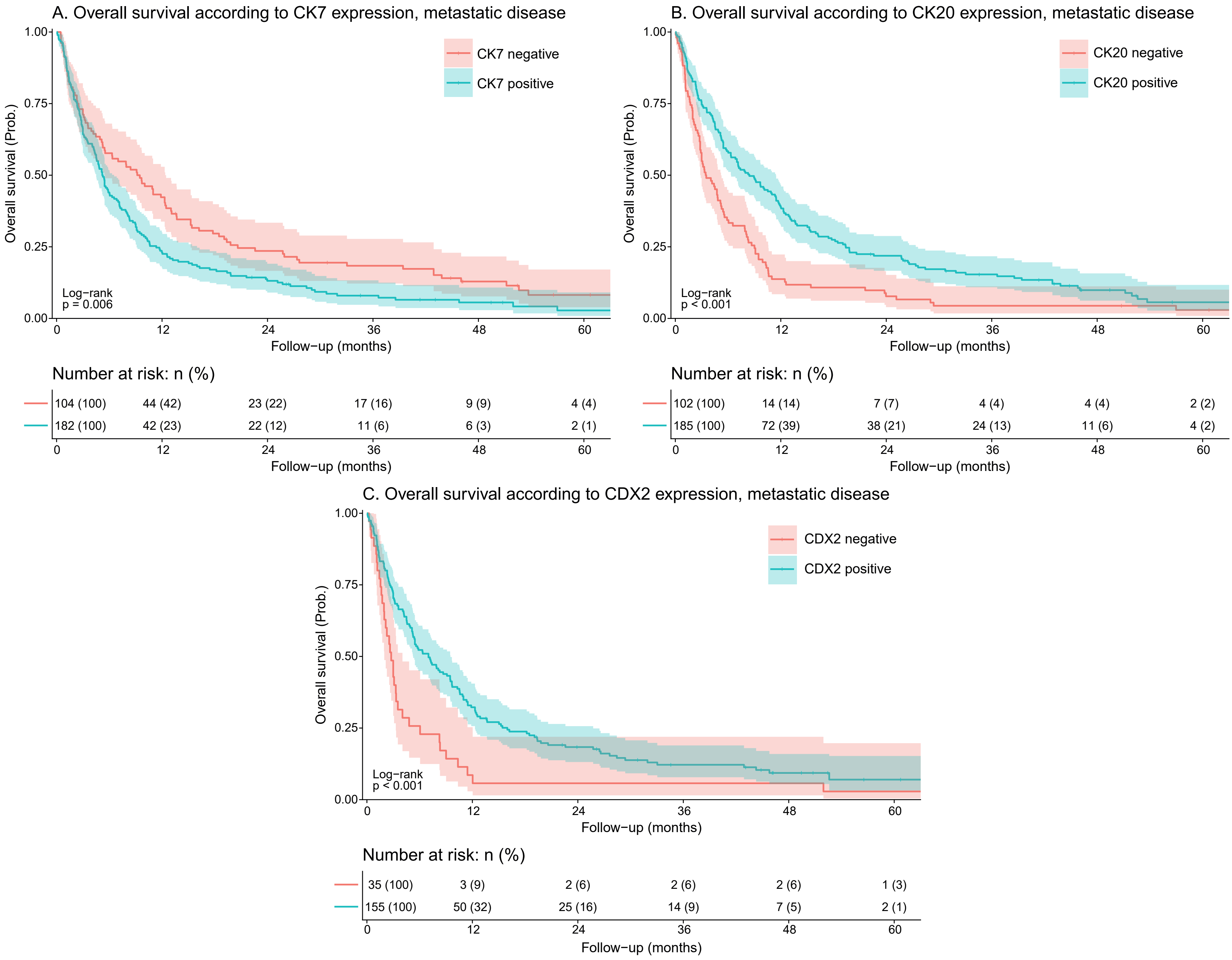

Supplementary figure 6

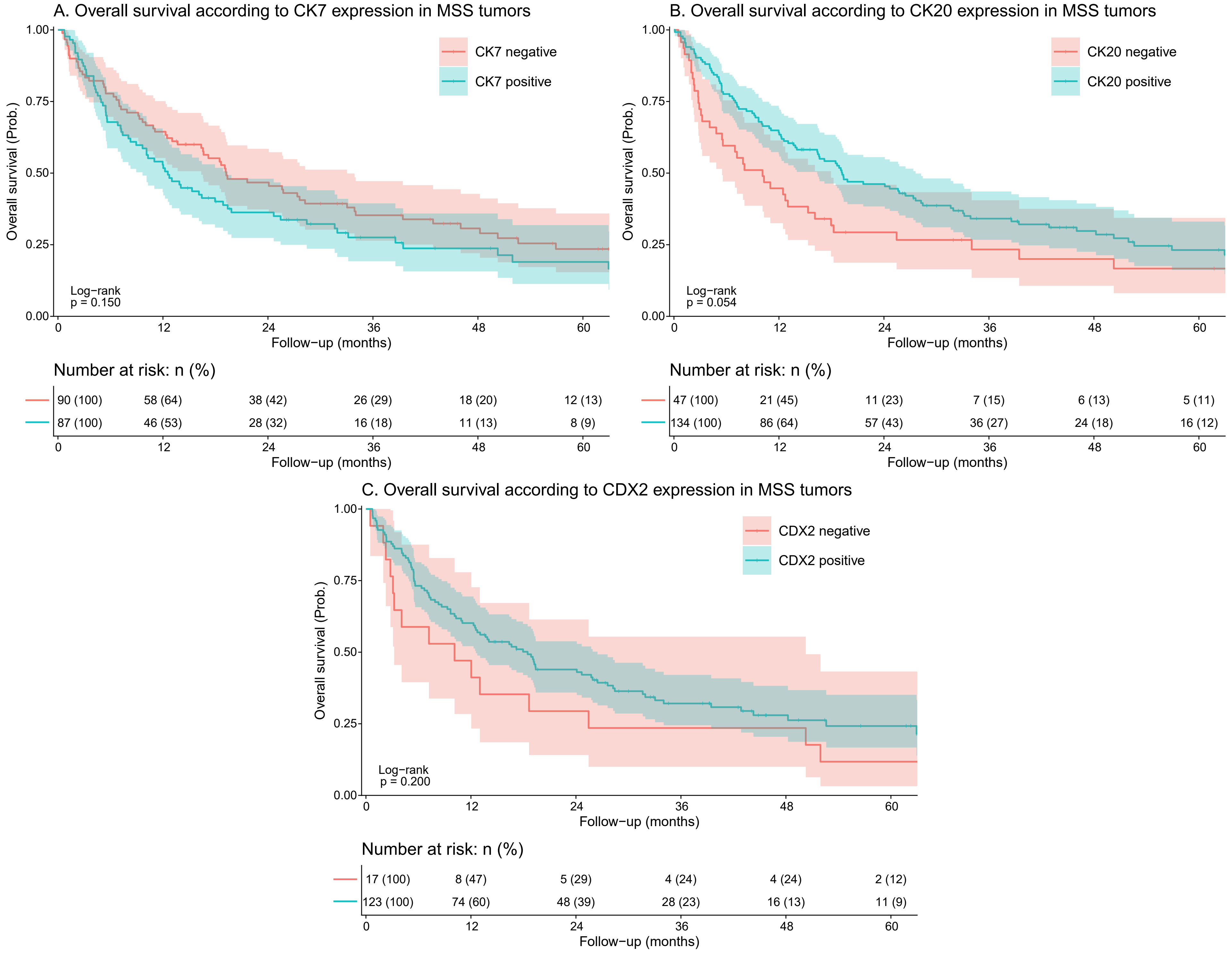

Supplement: Supplementary file 1 [file mmc1.pdf]
