# Supplementary material for: Prognostic significance of CK7, CK20, and CDX2 expression in small intestinal adenocarcinoma
Source: ESMO Gastrointest Oncol. 2026 Jun 18;13(Pt B):100346. doi: 10.1016/j.esmogo.2026.100346 (PMC13712183; doi:10.1016/j.esmogo.2026.100346)
Supplement: Supplementary file 2 [file mmc2.docx]

| **Supplementary table 1.** Registered expression profiles | |  |
| --- | --- | --- |
|  | Total NCR cohort  (n = 691)  n (%) | Total validation cohort  (n = 128)  n % |
| CK7 expression known  Positive*  Negative* | 651 (94)  369 (57)  282 (43) | 128 (100)  28 (22)  100 (78) |
| CK20 expression known  Positive*  Negative* | 647 (94)  429 (66)  218 (34) | 128 (100)  62 (48)  66 (52) |
| CDX2 expression known  Positive*  Negative* | 451 (65)  375 (83)  76 (17) | 128 (100)  84 (66)  44 (34) |
| CK7 & CK20 expression known  CK7-, CK20-*  CK7+, CK20-*  CK7-, CK20+*  CK7+, CK20+* | 626 (91)  60 (10)  151 (24)  215 (34)  200 (32) | 128 (100)  42 (33)  24 (19)  58 (45)  4 (3) |
| CK7 & CDX2 expression known  CK7-, CK20-*  CK7+, CK20-*  CK7-, CK20+*  CK7+, CK20+* | 423 (61)  19 (5)  54 (13)  172 (41)  178 (42) | 128 (100)  23 (18)  21 (16)  77 (60)  7 (6) |
| CK20 & CDX2 expression known  CK20-, CDX2-*  CK20+, CDX2-*  CK20-, CDX2+*  CK20+, CDX2+* | 423 (61)  59 (14)  13 (3)  87 (21)  264 (62) | 128 (100)  35 (27)  9 (7)  31 (24)  53 (41) |
| All expression profiles known  CK7-, CK20-, CDX2-*  CK7-, CK20+, CDX2-*  CK7-, CK20-, CDX2+*  CK7-, CK20+, CDX2+*  CK7+, CK20-, CDX2-*  CK7+, CK20+, CDX2-*  CK7+, CK20-, CDX2+*  CK7+, CK20+, CDX2+* | 414 (60)  13 (3)  5 (1)  34 (8)  135 (33)  46 (11)  7 (2)  52 (13)  122 (30) | 128 (100)  16 (13)  7 (6)  26 (20)  51 (40)  19 (15)  2 (2)  5 (4)  2 (2) |
| * Percentage based on total of total known expression per marker | |  |

| **Supplementary table 2.** Characteristics of patients diagnosed with a primary malignancy of the small intestine per combination of CK7, CK20 and CDX2 expression (NCR). | | | | | | | | | | | | | | | | | | | | | |  |  |  |
| --- | --- | --- | --- | --- | --- | --- | --- | --- | --- | --- | --- | --- | --- | --- | --- | --- | --- | --- | --- | --- | --- | --- | --- | --- |
|  | CK7-, CK20-, CDX2-  (n=13)  n (%) | | CK7-, CK20+, CDX2-  (n=5)  n (%) | | CK7-, CK20-, CDX2+  (n=34)  n (%) | | CK7-, CK20+, CDX2+  (n=135)  n (%) | | CK7+, CK20-, CDX2-  (n=46)  n (%) | | CK7+, CK20+, CDX2-  (n=7)  n (%) | | CK7+, CK20-, CDX2+  (n=52)  n (%) | | CK7+, CK20+, CDX2+  (n=122)  n (%) | | *p-value* | | | | | | | |
| Sex | |  | |  | |  | |  | |  | | | |  | |  |  | | 0.643 | | | |  |  |
| Male | 6 (46) | | 1 (20) | | 18 (53) | | 70 (52) | | 24 (52) | | 5 (71) | | 31 (60) | | 59 (48) | |  | | | |  |  |  |  |
| Female | 7 (54) | | 4 (80) | | 16 (47) | | 65 (48) | | 22 (48) | | 2 (29) | | 21 (40) | | 63 (52) | |  | | | |  |  |  |  |
| Age category at diagnosis | |  | |  | |  | |  | |  | | | |  | |  |  | | 0.395 | | | |  |  |
| Median [IQR] | 70 [66-73] | | 65 [57-74] | | 66 [59-71] | | 68 [58-75] | | 67 [60-75] | | 68 [57-78] | | 70 [61-77] | | 68 [59-78] | |  | | | |  |  |  |  |
| <50 | - | | 1 (20) | | 3 (9) | | 19 (14) | | 1 (2) | | - | | 4 (8) | | 14 (12) | |  | | | |  |  |  |  |
| 50-75 | 12 (92) | | 3 (60) | | 24 (71) | | 84 (62) | | 34 (74) | | 5 (71) | | 34 (65) | | 71 (58) | |  | | | |  |  |  |  |
| >75 | 1 (8) | | 1 (20) | | 7 (21) | | 32 (24) | | 11 (24) | | 2 (29) | | 14 (27) | | 37 (30) | |  | | | |  |  |  |  |
| Period of diagnosis | |  | |  | |  | |  | |  | | | |  | |  |  | | 0.073 | | | |  |  |
| 1999-2006 | - | | 1 (20) | | - | | 2 (1) | | 2 (4) | | 1 (14) | | 1 (2) | | 2 (2) | |  | | | |  |  |  |  |
| 2007-2013 | 4 (31) | | 3 (60) | | 8 (24) | | 33 (24) | | 15 (33) | | 3 (43) | | 12 (23) | | 36 (30) | |  | | | |  |  |  |  |
| 2014-2019 | 9 (69) | | 1 (20) | | 26 (76) | | 100 (74) | | 29 (63) | | 3 (43) | | 39 (75) | | 84 (69) | |  | | | |  |  |  |  |
| Tumor location | |  | |  | |  | |  | |  | | | |  | |  |  | | 0.052 | | | |  |  |
| Duodenum | 9 (69) | | 3 (60) | | 20 (59) | | 67 (50) | | 26 (57) | | 4 (57) | | 40 (77) | | 84 (69) | |  | | | |  |  |  |  |
| Jejunum | - | | 2 (40) | | 12 (35) | | 40 (30) | | 8 (17) | | 3 (43) | | 6 (12) | | 19 (16) | |  | | | |  |  |  |  |
| Ileum | 3 (23) | | - | | - | | 17 (13) | | 9 (20) | | - | | 3 (6) | | 12 (10) | |  | | | |  |  |  |  |
| Overlapping | 2 (8) | | - | | 1 (3) | | 2 (1) | | 1 (2) | | - | | 1 (2) | | 3 (3) | |  | | | |  |  |  |  |
| NOS | - | | - | | 1 (3) | | 9 (7) | | 2 (4) | | - | | 2 (4) | | 4 (3) | |  | | | |  |  |  |  |
| Stage | |  | |  | |  | |  | |  | | | |  | |  |  | | <0.001 | | | |  |  |
| I | 1 (8) | | 1 (20) | | 4 (12) | | 2 (2) | | - | | - | | - | | 2 (2) | |  | | | |  |  |  |  |
| II | 3 (23) | | 3 (60) | | 10 (30) | | 35 (28) | | 11 (25) | | 1 (25) | | 8 (17) | | 27 (24) | |  | | | |  |  |  |  |
| III | 5 (39) | | 1 (20) | | 8 (24) | | 38 (30) | | 9 (21) | | - | | 11 (24) | | 33 (29) | |  | | | |  |  |  |  |
| IV | 4 (31) | | - | | 11 (33) | | 52 (41) | | 24 (55) | | 3 (75) | | 27 (59) | | 52 (46) | |  | | | |  |  |  |  |
| *Missing* | *-* | | *-* | | *1* | | *8* | | *2* | | *3* | | *6* | | *8* | |  | | | |  |  |  |  |
| Grade of differentiation | |  | |  | |  | |  | |  | | | |  | |  |  | | 0.009 | | | |  |  |
| Well differentiated | 1 (11) | | - | | 2 (9) | | 2 (2) | | - | | 1 (17) | | 3 (9) | | 3 (4) | |  | | | |  |  |  |  |
| Moderately differentiated | 3 (33) | | 2 (50) | | 5 (22) | | 56 (64) | | 10 (31) | | 1 (17) | | 15 (46) | | 39 (50) | |  | | | |  |  |  |  |
| Poorly differentiated | 5 (56) | | 2 (50) | | 16 (70) | | 29 (33) | | 22 (69) | | 4 (67) | | 15 (46) | | 36 (46) | |  | | | |  |  |  |  |
| *Missing* | *4* | | *1* | | *11* | | *48* | | *14* | | *1* | | *19* | | *44* | |  | | | |  |  |  |  |
| Site of metastasesˆ | |  | |  | |  | |  | |  | | | |  | |  |  | | 0.820 | | | |  |  |
| Single | 3 (23) | | - | | 4 (36) | | 31 (60) | | 17 (71) | | 2 (67) | | 16 (59) | | 34 (65) | |  | | | |  |  |  |  |
| Two | 1 (8) | | - | | 3 (27) | | 17 (33) | | 5 (21) | | 1 (33) | | 9 (33) | | 14 (27) | |  | | | |  |  |  |  |
| Three or more | - | | - | | 3 (27) | | 4 (8) | | 2 (8) | | - | | 2 (7) | | 5 (10) | |  | | | |  |  |  |  |
| Overall survival (months) | |  | |  | |  | |  | |  | | | |  | |  |  | <0.001 | | <0.001 | | | |  |
| Median [95% CI] | 7.2 [2.8-NA] | | 50.3 [18.6-NA] | | 18.0 [9.5-NA] | | 20.6 [16.2-38.6] | | 3.8 [3.0-8.2] | | 4.8 [1.6-NA] | | 7.0 [3.3-10.6] | | 13.4 [8.3-23.6] | |  | | | |  |  |  |  |
| Abbreviations: IQR; interquartile range, NOS; not otherwise specified, 95% CI; 95% confidence interval  * Percentages may not equal 100 due to rounding.  ^ Percentages are based on the number of patients with registered metastases. | | | | | | | | | | | |  | | | | | | | | | |  |  |  |
